# A Cardiac Troponin T Biosensor Based on Aptamer Self-assembling on Gold

**DOI:** 10.22088/IJMCM.BUMS.8.4.271

**Published:** 2019

**Authors:** Masoud Negahdary, Mostafa Behjati-Ardakani, Hossein Heli, Naghmeh Sattarahmady

**Affiliations:** 1 *Nanomedicine and Nanobiology Research Center, Shiraz University of Medical Sciences, Shiraz, Iran.*; 2 *Yazd Cardiovascular Research Center, Shahid Sadoughi University of Medical Sciences, Yazd, Iran.*; 3 *Department of Medical Physics, School of Medicine, Shiraz University of Medical Sciences, Shiraz, Iran.*

**Keywords:** Myocardial infarction, aptasensing, biomarker, bioelectrochemical detection

## Abstract

In this study, a sensitive and accurate aptasensor was designed for early detection of myocardial infarction through the determination of troponin T (TnT). The successful immobilization of a specific aptamer sequence on the surface of gold that had a high affinity toward TnT was accomplished. TnT was electrochemically quantified. The results indicated that the aptasensor detected TnT in a range of 0.05-5 ng mL, and with a detection limit of 0.01 ng/mL. The performance of the aptasensor was investigated by analyzing 99 human serum samples. Both diagnostic specificity and sensitivity of the aptasensor were found to be 95%. The use of the designed aptamer-based biosensor could be an essential achievement in health policy, preventing deaths caused by myocardial infarction, and reducing patients with heart failure. The extensive use of this aptamer-based biosensor can also reduce costs, enhance speed, and improve accuracy in the diagnosis of TnT as an important myocardial infarction biomarker.

Myocardial infarction occurs when the bloodstream in the coronary arteries is cut through a clot (1). A broken bloodstream that occurs during a heart attack can damage or destroy some parts of the myocardium (2). Despite all the progress in medical sciences and the facilitation of the diagnosis and treatment of cardiovascular diseases, unfortunately, their prevalence is still rising in the world (3, 4). Traditional techniques do not show the required efficiency because of their time- consuming nature, and low accuracy (5). Also, the electrocardiography does not provide a detailed and exact diagnosis (6). Therefore, researchers have invented routes to find faster and more accurate results (7). If correct, timely, and appropriate measures are taken (in less than two hours after myocardial infarction), heart attack does not necessarily lead to death.

Biomarkers can be used for specific diagnosis of various diseases. Different types of molecules, such as DNA, genes, proteins, and hormones, can serve as a biomarker (8). An increase or decrease in the concentration of a biomarker in the body fluids can be directly related to the severity of a disease. However, some biomarkers are not only related to a specific disease, and may be found based on the damages that occurred in several organs or tissues. Cardiac biomarkers are released into the bloodstream from the damaged cardiac myocytes. Since even mild myocyte damages lead to an increase in the level of cardiac biomarkers (9), using these biomarkers is very useful in the rapid diagnosis of myocardial infarction, and reduction of its complications. The most critical and specific biomarker for the rapid detection of myocardial infarction is troponin (10). Troponin is a protein that controls the relationship between actin and myosin, which plays an essential role in contractions of various muscles, including the heart muscle (myocard). This protein is a complex that contains three subunits consisting of troponin C (TnC), troponin T (TnT), and troponin I (TnI) (11). Normally, the troponin level in the bloodstream is insignificant or indistinguishable. However, when the myocard is harmed, troponin is released into the bloodstream, and its concentration in the blood will increase; in this case, the more the damage to the myocard, the higher the blood troponin concentration will be (12). So far, most of the laboratory diagnostic methods for TnT assessment have been based on immunological assays (13, 14).

Biosensors are promising tools in clinical and biomedical researches. They are potential alternatives for the routine bioanalysis methods and systems because of their simplicity and ability to analyze complex matrices. Biosensors for biomarkers detection are alternative tools for disease diagnostics with high sensitivity and specificity (15-17). For biomarker detection, the selection of the biosensor’s bioreceptor is the troublesome primary step.

As promising tools, electrochemical biosen-sors have been developed for application in quality control, clinical diagnosis, biomedical researches, and point-of-care testing (15-19). Due to integrability with multi-arrays, miniaturizability, easy fabrication, rapid responses, low cost, simplicity, and sensitivity, these biosensors have become alternatives for disease detection (15-17, 19).

Using aptamers in the design of biosensors (aptasensors) has led to an increment in the specificity of the biosensors to capture the biomolecules (20). Aptasensors have relatively low cost and high binding affinity, and provide rapid diagnostic performance with high reproducibility (15, 17, 19). Compared to antibodies, aptamers have the advantage of easy production and biostability, and provide high selective biosensors applicable in cellular studies (21). Employment of aptamers in electrochemical biosensors has considerable attention because the combination of aptamer selectivity and electrochemical detection sensitivity makes the electrochemical aptasensors attractive tools for biological sample analysis (16-19). Therefore, more studies on electrochemical aptasensors are recommended.

Up to now, some electrochemical biosensors have been designed for the detection of TnT, and most of them have been based on immunological methods using antibodies (22-28). In this research, a specific aptamer with a high affinity against TnT was used to fabricate an aptasensor to quantify TnT in biological samples.

## Materials and methods

**Preparation of the TnT aptasensor**

The polishing surface of the gold disk electrode was followed on a pad inoculated with 0.05 m-alumina powder and lubricated with water. Polishing was continued till a mirror-face surface was obtained. To remove the alumina particles, the gold disk electrode was immersed in a 1:3 water/ethanol mixture, and sonicated in an ultrasound bath for 8 min. All solutions and dilutions were performed using deionized water. In order to immobilize the ready aptamer on the gold electrode surface, 10 µL of a dithiothreitol (DTT) (Sigma, USA) solution (500 mM, pH 5.2) containing 10 mM sodium acetate was added to a 40-mer thiolated DNA aptamer stock solution (5’-(SH)-(CH_2_)_6_-CG TG CA GT AC GC CA AC CT TT CT CA TG CG CT GC CC CT CT TA-3’, Bioneer Co, Korea). After 20 min, the mixture was extracted with 100 µL ethyl acetate (Sigma, USA) three times, whichever the upper layer was discarded. In the next step, 10 mM phosphate buffer solution containing 5 mM NaCl, 2 mM KCl, and 1mM MgCl_2_, pH 7.4 was added to achieve a 10 µM aptamer solution. To find the optimized time of aptamer immobilization, open circuit potential (OCP) was measured by a digital voltmeter of Mastech MS8340B (China) and screen-printed gold electrodes of DropSens (Spain). 10 μL of this solution was dropped on the surface of the gold electrode, and the immobilization of aptamer was processed at 4 C for the optimized immobilization time of 70 min (vide infra). In the last step, to cover the free surface and alignment of the aptamer strands, 10 μL 6-mercapto-1-hexanol (MCH) (Sigma,USA) 1.0 mM was dropped on the surface, and maintained for 30 min at room temperature. The aptasensor was washed with deionized water, and was ready for use.

**Detection of TnT**

The aptasensor binding time for capturing the TnT molecules was followed at 37 C. All electrochemical measurements were performed using a - Autolab potentiostat/ galvanostat equipped with GPES 4.9 software (the Nether-lands). A three-electrode system was used where a gold disk electrode, a platinum rod, and an Ag/AgCl, 3 M KCl electrode were applied as working, counter and reference electrodes, respectively. This attempt was evaluated using a fixed concentration (0.5 ng/mL) of human cardiac TnT (Sigma,USA) at various binding times of 7, 11, 15, 19, 23, 27 and 31 min, and the related differential pulse voltammograms (DPVs) were recorded. The maximum decrement of the current was considered as the optimum time for binding between aptamer and TnT. DPVs were recorded in an electrolyte solution containing Tris-HCl buffer (20 mM) + KCl (0.5 M) + ferro/ferricyanide redox marker (0.5 M). TnT was determined by the aptasensor in concentrations of 0.05, 0.125, 0.25, 0.5, 1.0, 2.0 and 5.0 ng/mL.

**Biological samples analysis**

Human serum samples (2 mL) were provided from 99 individuals (49 men and 50 women samples). and divided into four groups, including healthy (41), patient (48), renal failure (5), and liver disease (5). For human sampling, informed consent was received by the volunteers (for healthy samples) or patients, and all protocols were performed based on the guidelines of the Ethics Committee of the Shiraz University of Medical Sciences (license number 21194). The proposal for this research has been reviewed and approved by the Ethics Committee of Shahid Sadoughi University of Medical Sciences and Health Services, Yazd (code of ethics of IR. MEDICINE. REC.1396.238).

To check the probable false-positive results obtained from the aptasensor, the renal failure and liver disease samples were analyzed. The patients samples were already and separately confirmed by an enzyme-linked immunosorbent assay, using an ELISA Kit.

**ELISA method**

First, the 96-well ELISA kit (Monobind, USA) and other related contents and human samples were placed at room temperature for half an hour to reach room temperature. There were six standard vials with concentrations of 0, 1, 3, 6, 15, and 30 ng/mL. Standard vials were used as calibrators. The process of reading the optical density (OD) and sample concentration (at 450 nm wavelength and reference wavelength: 630 nm) was performed by an ELISA reader. A total of 89 human serum samples of heart attack (48) and healthy (41) subjects were classified according to the ELISA method report. The serum samples of patients with a heart attack were then diluted to the nearest standard of TnI (0.05 to 500 ng/mL). Then, the concentrations prepared along with the standard concentrations were measured by the aptamer- based biosensor. The serum samples of 5 kidney patients and 5 liver patients were also examined.

**Data analysis**

Data analysis was performed using GPES software version 4.9 and Excel software (2010).

## Results

**Aptamer characterization and optimization of the OCP**

**Fig. 1:**
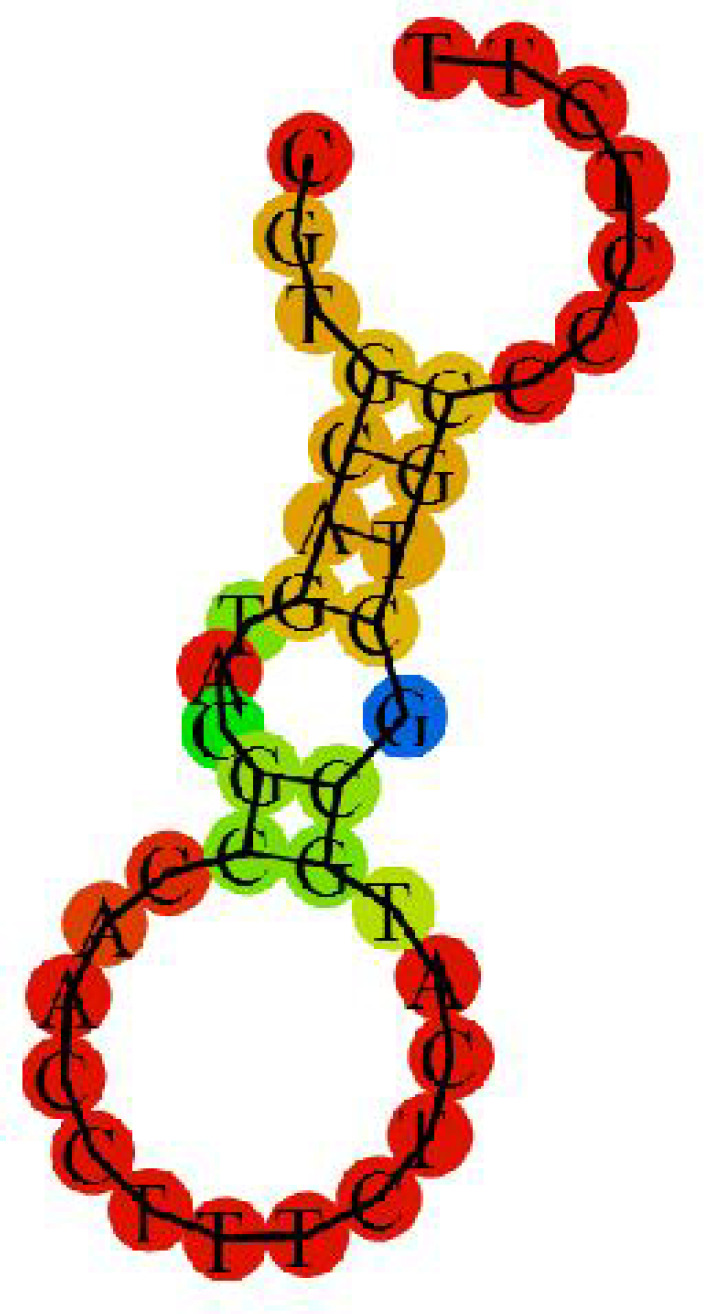
Secondary structure of the troponin T aptamer

Aptamers bind and recognize the protein targets through the secondary structure, folding, and 3D shapes of the aptamers as well as specific binding sites and different types of non-covalent attractions. There is a need for high avidity, affinity, specificity, and selectivity for the binding of aptamer-protein for an ultimate specific and sensitive biosensing. The secondary structure of the aptamer is shown in Figure 1.

**Fig. 2:**
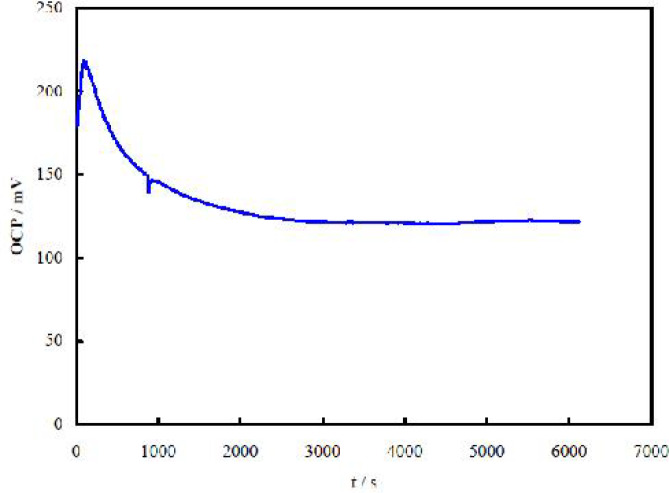
Changes in the OCP values during immobilization of the aptamer on the gold electrode surface

For inspection of the best time of aptamer immobilization on the gold surface to attain a maximum surface concentration of the (immob-ilized) aptamer, OCP measurements were followed. Upon self-assembling of thiols on gold surfaces, OCP value remains within the potentials of gold stability (29), and therefore, thiols binding on gold is performed without using a potential external application. OCP is determined with several parameters including change in the surface state upon immobilization of a charged species, and reaching a steady value for OCP is an indication of immobilization completion. The aptamer has a net negative charge arising from its phosphate groups, and its approach to the surface leads to changes in OCP. Changes in the OCP values during immobilization of the aptamer on the gold electrode surface are shown in Figure 2.

OCP values showed rapid changes toward positive values (at times shorter than 130 s) due to breaking of the pre-formed double-layer structure alongside the aptamer approach to the surface, and then (at times longer than 130 s) approached to negative values due to formation of a monolayer of the aptamer. Later, rearrangement would occur to form a packed aptamer monolayer (30). Based on the data presented in Figure 2, 1.7 h was considered for the best time of the aptamer immobilization.

**Optimization of TnT binding time**

**Fig. 3:**
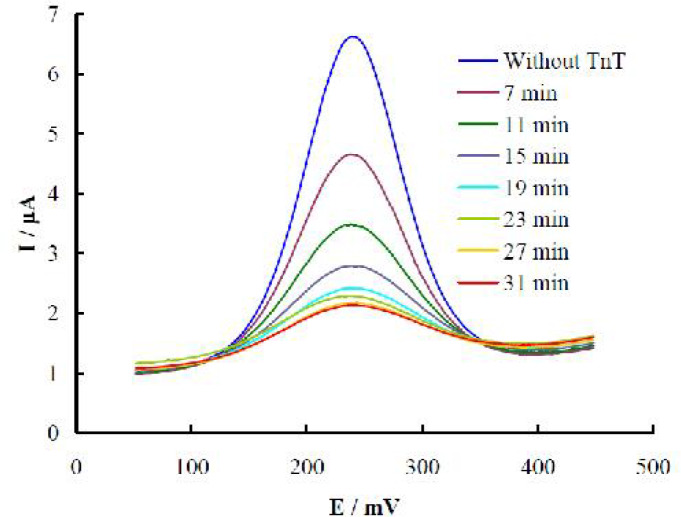
**DPVs recorded upon TnT incubation with the aptasensor at different binding times.** Several time points including before, and after 7, 11, 15, 19, 23, 27, and 31 min incubation were **recorded**

For evaluation of the optimized time of TnT binding with the aptasensor, 0.5 ng/mL TnT was exposed at various times at 37 C. DPVs recorded before and after TnT incubation with the aptasensor at different binding times are shown in Figure 3. 

**TnT aptamer-based biosensor design and detec-tion performance evaluation**

The peak in the voltammogram is due to the redox transition of the marker (ferro/ferricyanide), and the peak current was decreased upon prolonging the TnT binding. The marker had a certain approachability to the aptasensor surface that was determined by the repulsion forces between the negatively charged aptamer and marker. On the other hand, TnT bears a net negative charge arising from its deprotonation in the working 

**Fig. 4.**
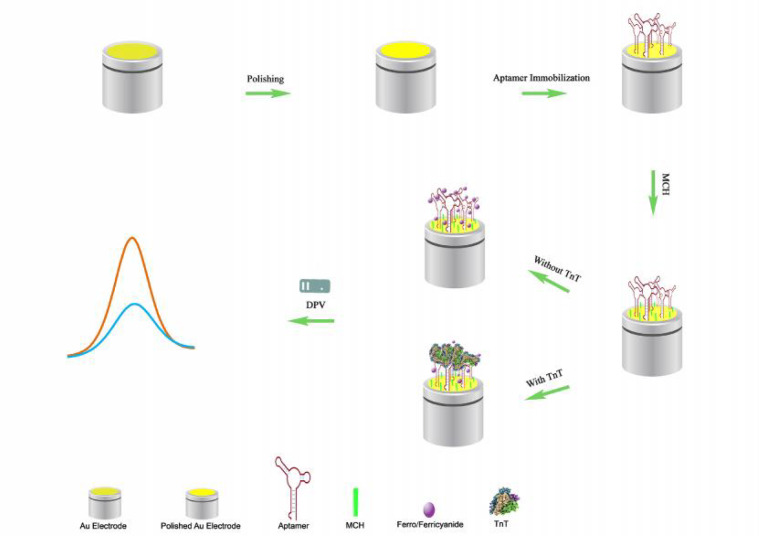
The fabrication and working steps of the troponin T aptasensor

pH (7.4) and its isoelectric point of 4.98-5.9 (31). Therefore, TnT binding with the aptamer increased the negative surface charge of the aptasensor leading to more repulsion of the marker and decrement in the peak currents. Based on the results presented in Figure 4, a binding (incubation) time of 30 min was considered as the best time. Based on these explanations, a diagrammatic represe-ntation of the fabrication steps and signal generation mechanism are also presented in Figure 4.

**Fig. 5.**
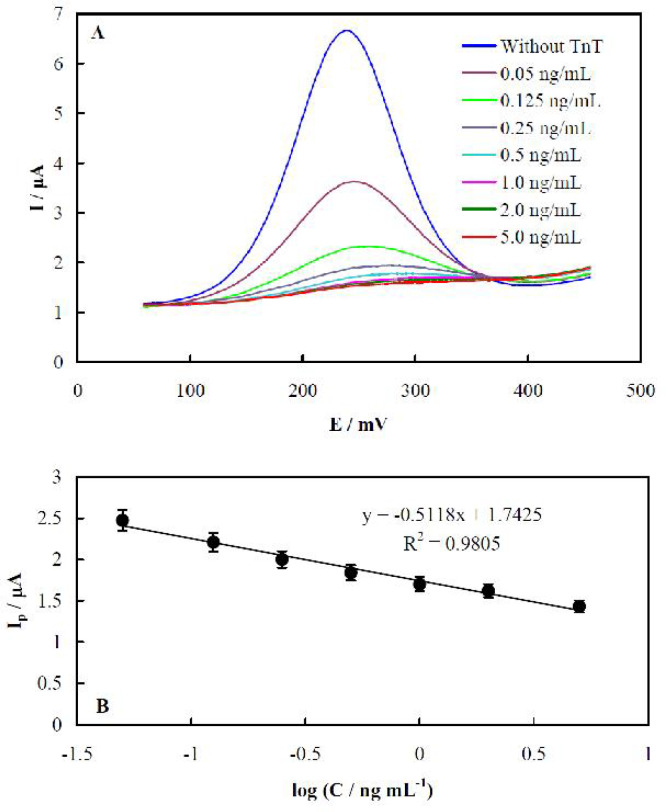
**Detection performance of the TnT aptasensor.** A: DPVs recorded using the aptasensor before and after binding with different TnT concentrations of 0.05, 0.125, 0.25, 0.5, 1.0, 2.0, and 5.0 ng/mL^-1^; B: a calibration plot for the dependency of the peak currents in panel A on the TnT concentration (the error bars refer to five times replicates)

In order to quantify TnT and find the linearity detection range of the aptasensor, various concentrations of TnT in a range of 0.05 to 5.0 ng/mL were assayed, and the resultant DPVs are shown in Figure 5A. The peak currents depended on the TnT concentration, and a calibration plot was constructed using the voltammograms, as displayed in Figure 5B.

The calibration plot represented a regression equation of I_p_ /A= -(0.5118.0.0323) log(C /ng/mL)+ (1.74250.0230/ A), R^2^ = 0.9805. Using the plot, we obtained a value of 0.01 ng/mL as a limit of detection (LOD, 3/X, is the standard deviation of the blank signal, and X is the calibration plot slope) of TnT for the aptasensor. In Table 1, various methods based on the Au transducers for the detection of TnT are presented and compared. In comparison to other TnT detection methods, the aptasensor is one of the limited TnT aptasensors, and represented a very low LOD value.

**Table 1:** A comparison between various methods of TnT detection based on the Au transducers

**Detection method**	**Transducer**	**Detection range /** **ng mL** ^-1^	**LOD/** **ng mL** ^-1^	**Real matrix**	**Ref.**
**Surface plasmon resonance** **immunosensing**	Au chip	0.03-6.5	0.01	Serum	(32)
**Surface plasmon resonance** **immunosensing**	Au disc	0.05-4.5	-	Serum	(33)
**Surface plasmon resonance** **immunosensing**	Au chip	<50000	100	-	(34)
**Magnetoimmunosensing**	Screen printed Au electrode	0.05-1.0	0.017	Serum	(25)
**Micro-fluxgate ** **immunosensing**	Au film-coated wafer	0.01-10	0.01	Serum	(35)
**Electrochemical ** **immunosensing**	Au electrode	10^-6^-100	0.0001	Serum	(36)
**Electrochemical ** **immunosensing**	Au electrode	0.1-10^3^	0.1	-	(37)
**Electrochemical ** **immunosensing**	Au electrode	0.1-10	0.033	Serum	(22)
**Amperometry**	Au electrode	0.05-1	0.017	Serum	(38)
**Electrochemical** **impedance spectroscopy**	Au electrode	0.001×10^-3^-10	1010^-6^	Plasma	(39)
**Electrochemical** **impedance spectroscopy**	Au electrode	73-1800	8.810^-6^	Whole blood	(40)
**Electrochemical** **impedance spectroscopy**	Au electrode	10^-5^-1	1010^-6^	Serum	(41)
**Electrochemical** **aptasensing**	Au electrode	0.05-5 ng mL^-1^	0.01	Serum	This study

**Reproducibility of the TnT aptasensor**

**Fig. 6.**
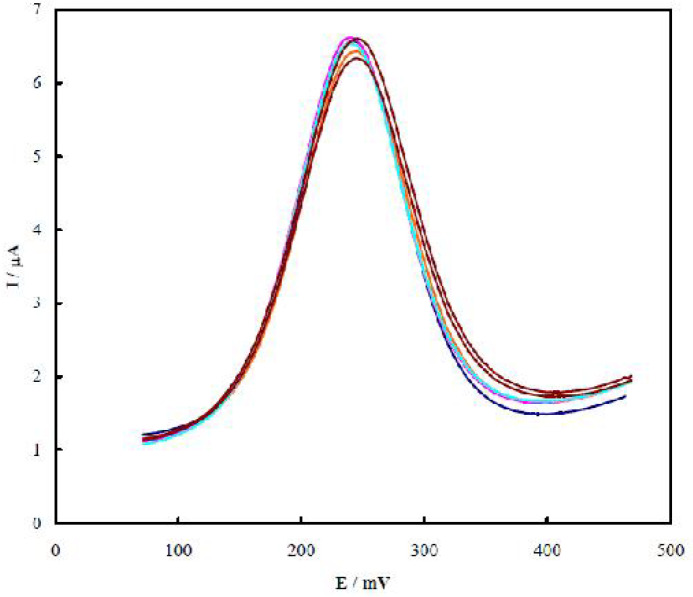
DPVs recorded using the aptasensor upon 6 times of preparation

In order to investigate the reproducibility of the TnT aptasensor fabrication, it was fabricated 6 times, and the related DPV for each time of fabrication was separately recorded. As shown in Figure 6, the current change in the voltammograms was very small, with a relative standard deviation (RSD) of 3.1%. This confirmed the regeneration ability of the aptasensor.

**Repeatability of the TnT aptasensor**

The repeatability of the TnT aptasensor to detect TnT was inferred by three independent measurements of TnT in one (an intra-day assay) or 3 days (an inter-day assay). The results showed RSD values lower than 4%. Besides, three determinations of 0.5 ng/mLTnT with a single aptasensor showed a RSD value of 3.5%.

**Evaluation of the TnT aptasensor regeneration**

**Fig. 7.**
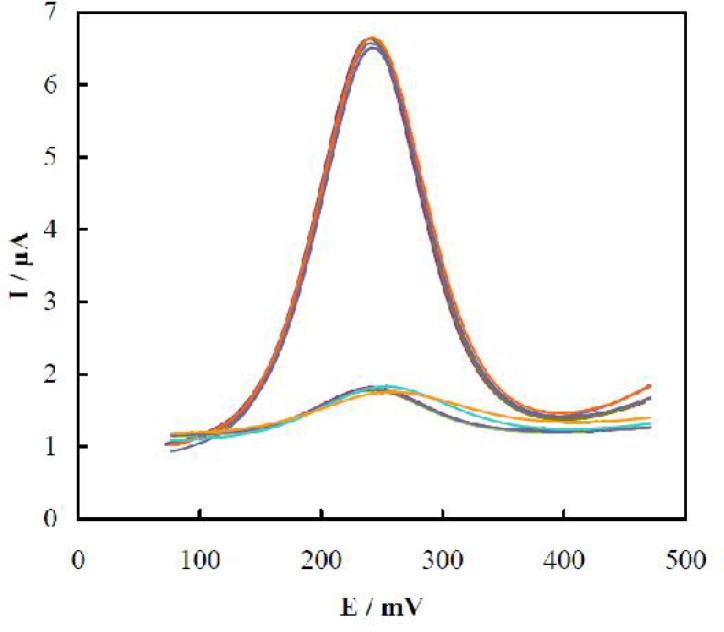
DPVs recorded using the aptasensor upon six times binding-unbinding of 0.5 ng/mL^-1^ TnT

The regeneration behavior of the TnT aptasensor was evaluated. This competency test was followed by six times binding-unbinding of 0.5 ng/mL TnT with the aptasensor, and the recorded pairs of DPVs after these cycles are shown in Figure 7. For removing the bound TnT, the aptasensor was placed in water at 95 C for 5 min, and washed with hot deionized water. The results showed excellent reusability for the aptasensor with RSD values of the peak current changes of 3.9 and 4.2% for TnT binding and unbinding, respectively.

**Evaluation of the TnT aptasensor stability**

The stability of the aptasensor was investi-gated upon binding with 0.5 ng/mL TnT, and recording DPVs during 13 days. The results indicated that the aptasensor signal was stable. during the first 12 days, and maintained 95% of its initial signal intensity

**Evaluation of the TnT aptasensor selectivity**

**Fig. 8:**
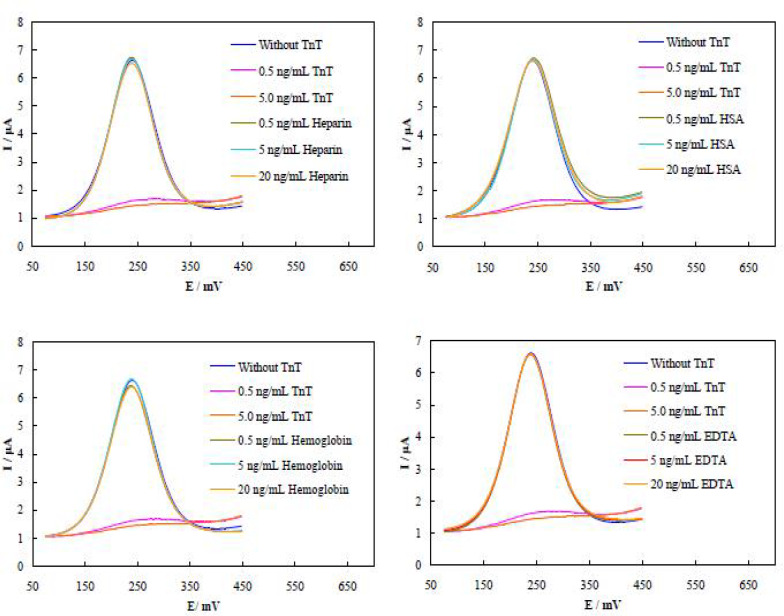
**DPVs measurement for binding of different biological compounds.** Binding of different concentrations of heparin, human serum albumin (HSA), hemoglobin, EDTA, and bilirubin was evaluated

To explore the selectivity of the TnT aptasensor, we assayed several interfering comp-ounds of heparin, human serum albumin (HSA), hemoglobin, ethylenediaminetetraacetic acid (EDTA), and bilirubin in the presence of TnT. The resultant DPVs are shown in Figure 8. 

Based on the recorded DPVs, the aptasensor was highly selective for detection of TnT with negligible interfering effect from the tested compounds.

**Biological samples evaluation**

To evaluate the applicability of the aptasensor, 99 human serum samples were assayed. Because the diagnosis limit level of TnT in the serum of a myocardial infarction patient is 0.1 ng/mL (42), aptasensor signal for 0.1 ng/mL TnT. It should be noted that the aptasensor is signal-off, and 10× is the signal of the limit of quantitation. The results of 99 human serum aptasensing and the comparison of the results of the immunoassay method are presented in Table 2. any aptasensor signal smaller than/equal to Y-10× is considered as positive, where Y is the The results depicted that the aptasensor could detect TnT in the serum samples with two false-positive and two false-negative outcomes. Accordingly, diagnostic sensitivity and specificity of the aptasensor were obtained as 95%.

## Discussion

Given the importance of myocardial infarction, the role of timely treatment, and prevention of complications, it can be said that the rapid and accurate diagnosis of this disease is urgent (1). People with specific symptoms of myocardial infarction usually undergo diagnostic and clinical evaluations to diagnose a heart attack. Unfortunately, one of the most important challenges is finding a way to quickly anticipate the specific risks posed by a heart attack (3, 4). Electr-ocardiograms do not show an accurate diagnosis of this heart disease in about half of the cases. Other diagnostic methods, such as echocardiography, angiography, etc. have a high cost, and also cannot be diagnosed quickly and in a short time. At present, the best way to diagnose a myocardial infarction is to quantify the heart biomarkers (8). The relationship between damage to heart myocytes and increased levels of cardiac biomarkers has been discovered for decades. Fast, simple, and accurate measurements of cardiac biomarkers are essential to confirm the occurrence of a heart attack. Cardiac troponin is the gold standard among cardiac biomarkers in the diagnosis of any damage to the myocardial tissue, and due to its superior selectivity, it has been widely used in the early detection of myocardial infarction (10). Electrochemical immunoassays are common methods for diagnosing troponin; however, these tools and methods are not portable and require steps that can only be performed in a clinical laboratory, and it is clear that these methods are inappropriate to quickly diagnose a case (13, 14). Achieving portable, low-cost, sensitive, and selective tools to diagnose myocardial infarction is important and unavoidable. Biosensors can be used as an important primary tool in diagnosing a heart attack, without hospitalization, at low cost, and in a short time. Biosensors are tools that respond to the presence of a specific analyte in an environment, and produce measurable signals (15-17). A biosensor consists of at least two components: a biorecognition element, and a transducer. Biosensors require a redox marker to detect the electrochemical behavior of various analytes. In this study, the redox marker was ferro/ferricyanide. This redox marker was chosen because of its ability to be easily redox, soluble in buffer solutions, non-binding exclusively to any surface that has a negative charge, and the ability to be close to different surfaces. In aptamer-based biosensors, an aptamer is used as the biorecognition element (20). Ideally, this component should have a high affinity (low detection limit), high specificity (minimum interference), wide dynamic range, and short response time. The signal transducer is responsible for converting the molecular changes to a measurable signal such as fluorescence, color creation, or electrical signals. The biophysical basis of aptamers against target molecules is related to the fact that these molecular structures can be folded as 3D structures. Aptamers always have a negative charge. The folding and creating loops are reducing the negative charge of aptamers (16-19). Aptamer strands can get as close to nanometers to the ferro/ferricyanide molecules. Amino acids have a positive charge in an acidic environment, and a negative charge in an alkaline environment. The binding of aptamer (negatively charged) to troponin (negatively charged) makes it more negative (reducing peak current) and repels ferro/ ferricyanide molecules, so the redox marker molecules are located in a far distance. In this study, a new, sensitive and selective aptamer-based biosensor was designed to diagnose cardiac TnT. Aptamer strands were immobilized on the surface of the gold electrode as the working electrode. The hybridization of TnT with the aptamer was electrochemically investigated. The choice of the gold electrode was for interaction with the thiolated aptamer. Gold and thiol provided a covalent Au-S bond, which is a very strong and stable chemical bond. Besides, this interaction is robust; it also conducts electricity. Rapid, accurate, specific, and low-cost detection are important advantages of the designed aptamer-based biosensor. For further research, it is recommended that the process of diagnosing other cardiac biomarkers be monitored using electrochemical methods. Various nanostr-uctures synthesized through biological processes can also be used to increase the sensitivity of these types of biosensors. In the field of increasing the accuracy of the diagnosis, it is also possible to increase the accuracy of the diagnosis to a greater level by calibrating the diagnostic tools. For further research, in addition to serum samples, plasma samples, whole blood, and urine samples are also recommended. People with unstable angina can also be included in the study groups. One of the main limitations of this study was the lack of comparison of the results obtained with available methods other than ELISA. The present aptasensor was applied to the analysis of biological samples, and is a promising tool to be employed for early diagnosis of myocardial infarction.

## References

[1] Mehta LS, Beckie TM, DeVon HA (2016). Acute Myocardial Infarction in Women: A Scientific Statement From the American Heart Association. Circulation.

[2] Mahrholdt H, Wagner A, Deluigi CC (2006). Presentation, patterns of myocardial damage, and clinical course of viral myocarditis. Circulation.

[3] Keates AK, Mocumbi AO, Ntsekhe M (2017). Cardiovascular disease in Africa: epidemiological profile and challenges. Nat Rev Cardiol.

[4] Townsend N, Nichols M, Scarborough P (2015). Cardiovascular disease in Europe--epidemiological update 2015. Eur Heart J.

[5] Benoit MO, Paris M, Silleran J (2001). Cardiac troponin I: its contribution to the diagnosis of perioperative myocardial infarction and various complications of cardiac surgery. Crit Care Med.

[6] Colaco R, Reay P, Beckett C (2000). False positive ECG reports of anterior myocardial infarction in women. J Electrocardiol.

[7] Allijn IE, Czarny BM, Wang X (2017). Liposome encapsulated berberine treatment attenuates cardiac dysfunction after myocardial infarction. J Control Release.

[8] Griffiths HR, Møller L, Bartosz G (2002). Biomarkers. Mol Aspects Med.

[9] Aldous SJ (2013). Cardiac biomarkers in acute myocardial infarction. Int J Cardiol.

[10] Reichlin T, Hochholzer W, Bassetti S (2009). Early diagnosis of myocardial infarction with sensitive cardiac troponin assays. N Engl J Med.

[11] Shettigar V, Zhang BY, Little SC (2016). Rationally engineered Troponin C modulates in vivo cardiac function and performance in health and disease. Nat Commun.

[12] Reichlin T, Irfan A, Twerenbold R (2011). Utility of absolute and relative changes in cardiac troponin concentrations in the early diagnosis of acute myocardial infarction. Circulation.

[13] Santi L, Farina G, Gramenzi A (2017). The HEART score with high-sensitive troponin T at presentation: ruling out patients with chest pain in the emergency room. Intern Emerg Med.

[14] Slagman A, von Recum J, Möckel M (2017). Diagnostic performance of a high- sensitive troponin T assay and a troponin T point of care assay in the clinical routine of an emergency department: a clinical cohort study. Int J Cardiol.

[15] Rahi A, Sattarahmady N, Heli H (2016). Label-free electrochemical aptasensing of the human prostate-specific antigen using gold nanospears. Talanta.

[16] Yazdani Z, Yadegari H, Heli H (2019). A molecularly imprinted electrochemical nanobiosensor for prostate specific antigen determination. Anal Biochem.

[17] Negahdary M, Heli H (2019). An ultrasensitive electrochemical aptasensor for early diagnosis of Alzheimer's disease, using a fern leaves-like gold nanostructure. Talanta.

[18] Chen A, Chatterjee S (2013). Nanomaterials based electrochemical sensors for biomedical applications. Chem Soc Rev.

[19] Akhtartavan S, Karimi M, Sattarahmady N (2020). An electrochemical signal-on apta-cyto-sensor for quantitation of circulating human MDA-MB-231 breast cancer cells by transduction of electro-deposited non-spherical nanoparticles of gold. J Pharm Biomed Anal.

[20] Tombelli S, Minunni M, Mascini M (2005). Analytical applications of aptamers. Biosens Bioelectron.

[21] Kun W, Zhan-Hui T, Lei X (2014). Research and development of functionalized aptamer based biosensor. Chin J Anal Chem.

[22] Gomes-Filho S, Dias A, Silva M (2013). A carbon nanotube-based electrochemical immunosensor for cardiac troponin T. Microchem J.

[23] Silva BV, Cavalcanti IT, Mattos AB (2010). Disposable immunosensor for human cardiac troponin T based on streptavidin-microsphere modified screen-printed electrode. Biosens Bioelectron.

[24] Karimian N, Turner AP, Tiwari A (2014). Electrochemical evaluation of troponin T imprinted polymer receptor. Biosens Bioelectron.

[25] de Ávila BEF, Escamilla‐Gómez V, Campuzano S (2013). Disposable electrochemical magnetoimmunosensor for the determination of troponin T cardiac marker. Electroanalysis.

[26] Zanato N, Talamini L, Zapp E (2017). Label‐free Electrochemical Immunosensor for Cardiac Troponin T Based on Exfoliated Graphite Nanoplatelets Decorated with Gold Nanoparticles. Electroanalysis.

[27] Liyanage T, Sangha A, Sardar R (2017). Achieving biosensing at attomolar concentrations of cardiac troponin T in human biofluids by developing a label-free nanoplasmonic analytical assay. Analyst.

[28] Zong C, Zhang D, Yang H (2017). Chemiluminescence immunoassay for cardiac troponin T by using silver nanoparticles functionalized with hemin/G-quadruplex DNAzyme on a glass chip array. Microchim Acta.

[29] Ma F, Lennox RB (2000). Potential-assisted deposition of alkanethiols on Au: controlled preparation of single-and mixed-component SAMs. Langmuir.

[30] Dijksma M, Kamp B, Hoogvliet JC (2000). Formation and electrochemical characterization of self-assembled monolayers of thioctic acid on polycrystalline gold electrodes in phosphate buffer pH 74. Langmuir.

[31] Iwańska E, Mikołajczak B, Grześ B (2016). Impact of post mortem aging of pork on changes in the isoelectric point of the proteins and tenderness. Med Weter.

[32] Dutra RF, Kubota LT (2007). An SPR immunosensor for human cardiac troponin T using specific binding avidin to biotin at carboxymethyldextran-modified gold chip. Clin Chim Acta.

[33] Dutra RF, Mendes RK, Lins da Silva V (2007). Surface plasmon resonance immunosensor for human cardiac troponin T based on self-assembled monolayer. J Pharm Biomed Anal.

[34] Liu JT, Chen CJ, Ikoma T (2011). Surface plasmon resonance biosensor with high anti-fouling ability for the detection of cardiac marker troponin T. Anal Chim Acta.

[35] Guo L, Yang Z, Zhi S (2017). Sensitive detection of cardiac troponin T based on superparamagnetic bead-labels using a flexible micro-fluxgate sensor. RSC advances.

[36] Shanmugam NR, Muthukumar S, Selvam AP (2016). Electrochemical nanostructured ZnO biosensor for ultrasensitive detection of cardiac troponin-T. Nanomedicine (Lond).

[37] Hsueh HT, Lin CT (2016). An incremental double-layer capacitance of a planar nano gap and its application in cardiac-troponin T detection. Biosens Bioelectron.

[38] Abad L, Javier del Campo F, Munoz FX (2012). Design and fabrication of a COP-based microfluidic chip: chronoamperometric detection of Troponin T. Electrophoresis.

[39] Shanmugam NR, Selvam AP, Barrett TW (2015). Portable nanoporous electrical biosensor for ultrasensitive detection of Troponin-T. Future Sci OA.

[40] Barrett TW, Radha Shanmugam N, Selvam AP (2015). Novel Nanomonitor ultra-sensitive detection of troponin T. Clin Chim Acta.

[41] Munje RD, Jacobs M, Muthukumar S (2015). A novel approach for electrical tuning of nano-textured zinc oxide surfaces for ultra-sensitive troponin-T detection. Anal Methods.

[42] Ohman EM, Armstrong PW, Christenson RH (1996). Cardiac troponin T levels for risk stratification in acute myocardial ischemia. N Engl J Med.

